# Impact of the Disc Vacuum Phenomenon on Surgical Outcomes in Lumbar Spinal Stenosis: A Comparative Study between Endoscopic Decompression and Minimally Invasive Oblique Lateral Interbody Fusion

**DOI:** 10.3390/jcm13195827

**Published:** 2024-09-29

**Authors:** Hyung Rae Lee, Kun Joon Lee, Seung Yup Lee, Jae Hyuk Yang

**Affiliations:** 1 Department of Orthopedic Surgery, Korea University Anam Hospital, Seoul 02855, Republic of Korea; drhrleeos@gmail.com (H.R.L.); yup1019@naver.com (S.Y.L.); 2 College of Medicine, Korea University, Seoul 02855, Republic of Korea; slcjohn@daum.net

**Keywords:** vacuum phenomenon, lumbar spinal stenosis, endoscopic decompression, MIS OLIF, surgical outcomes

## Abstract

**Objective**: This study investigated the influence of the vacuum phenomenon (VP) on surgical outcomes in patients with lumbar spinal stenosis, comparing minimally invasive oblique lateral interbody fusion (MIS OLIF) and endoscopic decompression. **Methods**: A cohort of 110 patients diagnosed with lumbar spinal stenosis underwent either endoscopic decompression or MIS OLIF. Patients were classified into two groups based on the presence or absence of the VP on preoperative CT scans, non-VP (n = 42) and VP (n = 68). Radiologic and clinical outcomes, including back and leg pain assessed using the visual analogue scale (VAS), the Oswestry Disability Index (ODI), and the EuroQol-5 Dimension (Eq5D), were compared pre- and postoperatively over a 2-year follow-up period. **Results**: Preoperatively, the VP group exhibited significantly greater leg pain (*p* = 0.010), while no significant differences were observed in back pain or the ODI between the groups. In the non-VP group, decompression and fusion yielded similar outcomes, with decompression showing a better ODI score at 1 month (*p* = 0.018). In contrast, in the VP group, patients who underwent fusion showed significantly improved long-term leg pain outcomes compared to those who underwent decompression at both 1-year (*p* = 0.042) and 2-year (*p* = 0.017) follow-ups. **Conclusions**: The VP may indicate segmental instability and may play a role in the persistence of radiculopathy. Fusion surgery appears to offer better long-term relief in patients with the VP, whereas decompression alone is a viable option in non-VP cases. These findings suggest that the VP may be a useful factor in guiding surgical decision-making.

## 1. Introduction

Lumbar spinal stenosis is prevalent in the ageing population and often necessitates surgical intervention when conservative treatments fail [1,2,3]. Traditionally, spinal surgeons rely on two main surgical options, spinal decompression and fusion [4]. However, recent advancements in minimally invasive techniques, particularly endoscopic decompression, have revived interest in decompression alone, offering benefits such as reduced surgical times and a quicker recovery [4,5,6,7,8]. Despite these advancements, the decision between decompression and fusion remains challenging, particularly in the presence of factors indicating instability, such as isthmic spondylolisthesis, dynamic instability, and facet diastasis [1,4,8]. In this context, the disc vacuum phenomenon (VP), which is indicative of instability at the disc level, has attracted considerable attention [6,9,10,11,12,13,14,15,16]. While traditionally associated with the management of back pain through procedures such as cement discoplasty, emerging research suggests that the VP may also serve as a key factor in determining the necessity for fusion surgery [9,17].

The VP is characterized by the accumulation of gas within the intervertebral disc, typically appearing as a radiolucent area on plain radiographs or as hypodense regions on CT scans [14,15,16]. Previous studies have demonstrated a correlation between the VP and increased back pain, as well as greater instability, particularly when vertical motion exceeds a certain threshold on dynamic radiographs [12,18,19]. The VP reflects disc instability, making it an essential consideration in surgical planning, particularly in cases where conservative treatments have failed to address symptoms. While these findings highlight the potential importance of the VP in surgical decision-making, the question remains as to whether the VP alone warrants fusion surgery or whether decompression could be equally effective.

There is no consensus on whether the VP should be considered a definitive indication for fusion akin to conditions such as isthmic spondylolisthesis [1,4]. Additionally, the long-term comparative outcomes of fusion versus decompression in patients with the VP have not been thoroughly investigated. While the VP is often viewed as a marker of instability, there are cases where patients with the VP can undergo endoscopic decompression alone and achieve favourable outcomes without fusion, suggesting that the VP does not always necessitate fusion surgery. This highlights the importance of evaluating each case individually, balancing the potential instability indicated by the VP against the benefits of a less invasive decompression procedure. Endoscopic decompression and MIS OLIF (minimal invasive oblique lateral interbody fusion) are effective surgical options; however, they operate based on different principles. MIS OLIF achieves indirect decompression by restoring disc height, which can alleviate symptoms without directly decompressing neural elements [20,21,22,23]. Over time, this approach may facilitate biological remodelling, potentially reversing degenerative changes and stabilizing the spine [20]. In contrast, endoscopic decompression directly targets neural elements but does not result in arthrodesis [2,5].

Therefore, this study aimed to compare the radiological and clinical outcomes of endoscopic decompression and MIS OLIF in patients with lumbar spinal stenosis, focusing on the presence and implications of the VP.

## 2. Materials and Methods

### 2.1. Study Design and Patients

**Figure 1:**
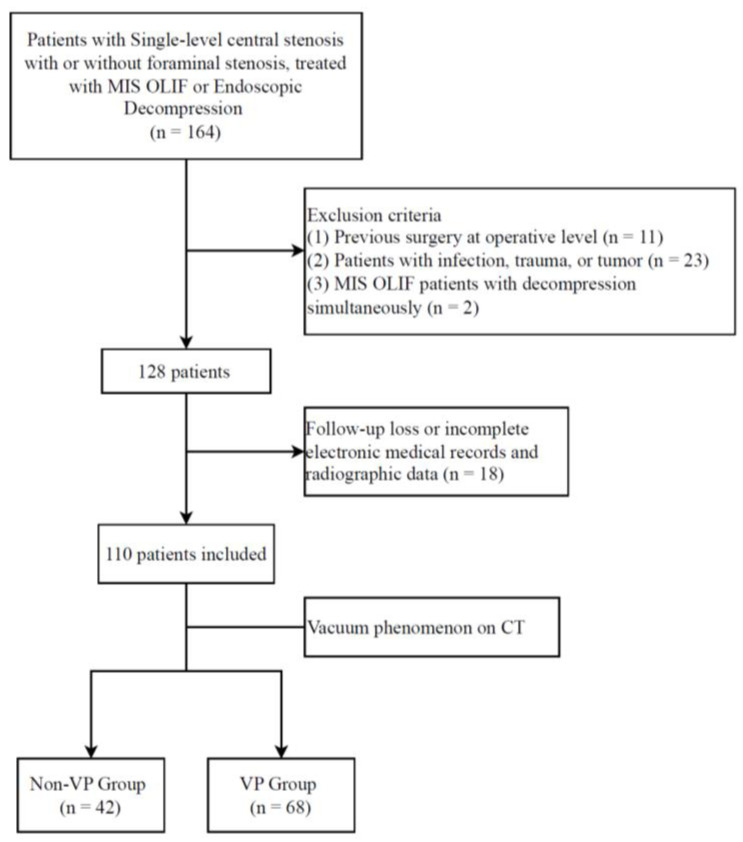
Patient selection flow chart. The figure outlines the process of including and excluding patients from the study cohort, from the initial screening to the final categorization into VP and non-VP groups.

This cohort study was approved by the Institutional Review Board (IRB No. 2024AN0320, approval date 2024-07-15). This study was conducted following the ethical principles of the Declaration of Helsinki and adhered to the Strengthening the Reporting of Observational Studies in Epidemiology guidelines to ensure transparency and rigour in the presentation of observational research [24]. The study population comprised patients diagnosed with single-level central lumbar stenosis, with or without foraminal stenosis, who underwent MIS OLIF or endoscopic decompression between January 2019 and June 2022. The patient selection process is illustrated in Figure 1. Initially, 164 patients with single-level central lumbar spine stenosis, with or without foraminal stenosis, were screened for inclusion and treated with MIS OLIF or endoscopic decompression. Patients included in the study presented with symptoms of neurogenic claudication and/or sciatica due to central stenosis or severe leg pain due to foraminal stenosis. The decision to proceed with surgical treatment was made after discussions with the patients, particularly when they did not achieve satisfactory symptom relief from conservative treatments, such as medication or nerve blocks. To meet the insurance criteria, patients undergoing decompression were required to have received at least 6 weeks of conservative treatment, and those undergoing fusion surgery needed a minimum of 3 months of conservative management. The diagnosis of lumbar stenosis was based on clinical symptoms compatible with radiological findings on MRI, where central or foraminal stenosis was observed. The inclusion criteria were restricted to single-level surgeries, meaning only patients requiring surgery at a single lumbar level were included in the study. The exclusion criteria were as follows: patients who had undergone previous surgery on the affected segment (n = 11); those with infections, trauma, or tumours affecting the lumbar spine (n = 23); and patients who underwent MIS OLIF concurrently with open or endoscopic decompression (n = 2). After applying these exclusion criteria, 128 patients were eligible for further analysis. An additional 18 patients were excluded due to loss of follow-up or incomplete electronic medical records and radiographic data, resulting in a final cohort of 110 patients. These 110 patients were subsequently categorized into two groups based on the presence of the VP in the disc space at the surgical level, as observed on CT scans. The non-VP and VP groups comprised 42 and 68 patients, respectively.

### 2.2. Surgical Procedures

The surgical procedures included biportal endoscopic interlaminar decompression and MIS OLIF.

Endoscopic decompression was performed via the interlaminar approach using the biportal endoscopic technique [2,25]. This procedure involves the creation of two small portals, one for the endoscope and another for the surgical instruments. Decompression was achieved by resecting the ligamentum flavum and performing a partial laminotomy. When necessary, the hypertrophic facet joints were trimmed to relieve nerve compression. The biportal approach allows adequate visualization and decompression of neural elements while minimizing soft tissue disruption. This procedure was performed by one of the authors (H.R.L). The MIS OLIF was performed with the patient in the lateral decubitus position [20,26,27]. An oblique approach was used to access the disc space anteriorly, avoiding the psoas muscle and reducing the risk of lumbar plexus injury. The intervertebral disc was removed, and an interbody cage was inserted to restore disc height and spinal alignment. Subsequently, percutaneous pedicle screw fixation was performed using a posterior approach to provide additional stability to the surgical segments. The OLIF procedures were conducted by only one senior author (J. H. Y.) to ensure consistency in the surgical technique across patients.

### 2.3. Data Collection and Radiologic Assessments

**Figure 2:**
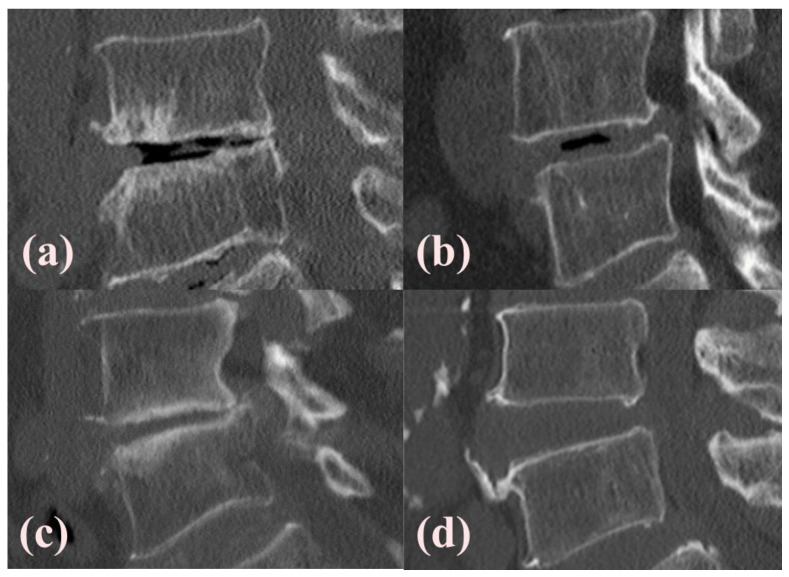
Illustration of the vacuum phenomenon (VP) and an endplate sclerosis assessment. The CT sagittal view shows the presence of the air in the disc space, indicating the VP. The most prominent VP cut was used for evaluation. Based on the ratio of the VP area to the disc area, the grades were categorized as follows: <20% as Grade 1, 20–80% as Grade 2, and >80% as Grade 3. Endplate sclerosis was noted when more than 20% of the vertebral endplate exhibited sclerotic changes. The VP with endplate sclerosis is shown in (**a**), the VP without endplate sclerosis in (**b**), non-VP with endplate sclerosis in (**c**), and non-VP without endplate sclerosis in (**d**).

Demographic characteristics such as age, sex, American Society of Anesthesiologists (ASA) classification, height, weight, body mass index (BMI), bone mineral density (BMD), and medical history (including hypertension, diabetes mellitus, and smoking status) were extracted from the patients’ electronic medical records. Additionally, details regarding the type and specific level of surgery were documented. Radiological measurements were performed to assess the structural characteristics of the lumbar spine. Instability in spondylolisthesis (ISL) was evaluated using plain and dynamic lateral lumbar radiography. Preoperative MRI axial cuts were used to assess the degree of central stenosis and graded according to the Schizas classification [28]. The presence and extent of the VP were determined through preoperative CT scans [11,14]. Based on the percentage of the disc space occupied by a vacuum (V), the VP was classified into four grades as follows: Grade 0 (no VP), Grade 1 (V < 20%), Grade 2 (20% ≤ V < 80%), and Grade 3 (V ≥ 80%), as illustrated in Figure 2. Additionally, endplate sclerosis was noted [22]. Quantitative measurements included anterior disc height (ADH), posterior disc height (PDH), foraminal height, and foraminal area. These measurements were obtained from preoperative CT scans using the region of interest (ROI) measurement function in a picture archiving and communication system (PetaVision for Clinics, 3.1, Korea University Anam Hospital, Seoul, Republic of Korea).

### 2.4. Clinical Outcome Measures

Clinical outcomes were evaluated preoperatively and at multiple postoperative time points for up to 2 years, including 1 month, 6 months, 1 year, and 2 years. The outcomes measured were back pain using the visual analogue scale (VAS), leg pain VAS, the Oswestry Disability Index (ODI), and the EuroQol-5 Dimension (Eq5D). These patient-reported outcome measures were collected to assess the effectiveness of surgical interventions in both the VP and non-VP groups.

### 2.5. Statistical Analyses

Statistical analyses were performed using the SPSS software (version 26.0; IBM Corp., Armonk, NY, USA). Continuous variables were expressed as mean ± standard deviation (SD), and categorical variables were presented as numbers and percentages. The Shapiro–Wilk test was used to assess the normality of continuous variables [29]. For comparisons between the VP and non-VP groups, an independent *t*-test was used for continuous variables, and the Chi-squared test or Fisher’s exact test was used for categorical variables, as appropriate. Repeated measures analysis of variance was used for normally distributed continuous variables to evaluate the clinical outcomes between decompression and fusion surgeries within each group, with post hoc pairwise comparisons performed using the Bonferroni correction. Statistical significance was set at *p* < 0.05.

Quantitative variables, including ADH, PDH, foraminal height, and foraminal area, as well as the VP grade, were independently measured by two spine surgeons who had completed their fellowship training. The surgeons were blinded to each other’s assessments and the patient groups. To ensure the reliability of the measurements, interobserver and intraobserver variabilities were calculated using the intraclass correlation coefficient (ICC).

## 3. Results

### 3.1. Demographic and Clinical Characteristics

Of the 110 patients, 42 were classified as non-VP and 68 were classified as VP. The VP group was slightly older (70.4 ± 9.0 vs. 67.5 ± 11.2 years, *p* = 0.203) with a similar male-to-female ratio (*p* = 0.165). ASA classification, height, weight, BMI, BMD, and the prevalence of hypertension, diabetes mellitus, and smoking were comparable between the groups (*p* > 0.05). The proportion of patients who underwent decompression or fusion was similar in both groups (*p* = 0.556). Additionally, there was no significant difference in the operative locations between the groups, with most surgeries performed at L4–5 (*p* = 0.616) (Table 1).

### 3.2. Imaging Characteristics

The Schizas grade, which reflects the severity of central stenosis, showed no significant difference (*p* = 0.773). In the VP group, the VP grades were classified as Grade 1 (39.7%), Grade 2 (42.6%), and Grade 3 (17.6%). Endplate sclerosis was significantly more prevalent in the VP group (48.5% vs. 14.3%, *p* = 0.001). Although ISL was slightly more frequently experienced in patients within the VP group (8.8% vs. 4.8%), this difference was not significant (*p* = 0.425). The VP group exhibited a shorter ADH (7.6 ± 2.8 mm vs. 9.7 ± 2.9 mm) and PDH (3.8 ± 1.4 mm vs. 5.5 ± 1.9 mm) (both *p* < 0.001), but foraminal measurements in terms of foraminal height and area showed no significant differences (*p* > 0.05) (Table 2). The reliability of these measurements was assessed using the ICC, which demonstrated excellent interobserver and intraobserver agreement. For ADH, the interobserver reliability using the ICC was 0.85 and the intraobserver reliability was 0.88. PDH had an interobserver reliability of 0.82 and an intraobserver reliability of 0.87. Similarly, the foraminal height and area had an interobserver reliability of 0.83 and 0.84 and an intraobserver reliability of 0.89 and 0.86. Furthermore, the VP grading showed the highest reliability, with an interobserver reliability of 0.96 and intraobserver reliability of 0.98.

### 3.3. Clinical Outcomes

In the preoperative assessment, the VP group (n = 68) demonstrated a greater mean leg pain on the VAS (5.3 ± 2.1) than that of the non-VP group (n = 42), which had a mean leg pain VAS score of 4.4 ± 1.8 (*p* = 0.010). However, no significant differences were observed between the two groups in terms of back pain (*p* = 0.55), the ODI (*p* = 0.335), or the Eq5D scores (*p* = 0.856). At the 2-year follow-up, there were no significant differences between the VP and non-VP groups in any of the assessed clinical outcomes, including back pain (*p* = 0.948), leg pain (*p* = 0.422), the ODI score (*p* = 0.085), and the Eq5D score (*p* = 0.449) (Table 3). 

When comparing the clinical outcomes between decompression and fusion within the non-VP group (Table 4), no significant differences were observed in back pain, leg pain, and the ODI and Eq5D scores across all assessed time points. However, at the 1-month follow-up, the fusion group had a significantly higher ODI score (44.3 ± 15.6) than that of the decompression group (33.0 ± 13.9), with a *p* value of 0.018. No other significant differences were noted between the two groups in the longer term.

**Figure 3:**
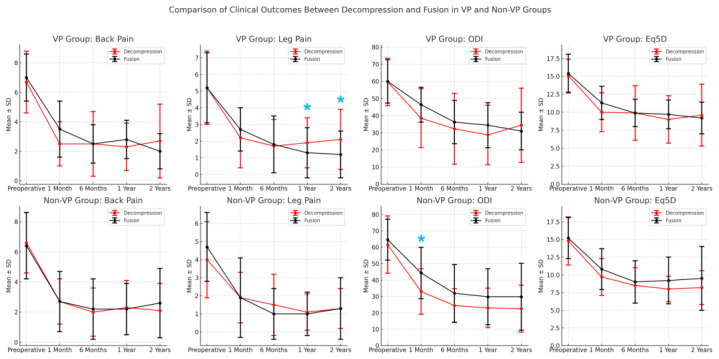
Comparison of clinical outcomes (back pain, leg pain, ODI, Eq5D) over time between decompression and fusion subgroups within the VP and non-VP groups. The blue asterisks (*) indicate points where *p* < 0.05.

In the VP group (Table 5), patients who underwent fusion (n = 36) showed significantly better outcomes in terms of leg pain at both the 1-year (*p* = 0.042) and 2-year (*p* = 0.017) follow-ups than those who underwent decompression (n = 32). Although the fusion group had lower ODI scores at various time points, these differences were not statistically significant. Similarly, the groups had no significant differences in back pain or Eq5D scores throughout the follow-up period. These results are illustrated in Figure 3, which presents the trends in the clinical outcomes (back pain, leg pain, and ODI and Eq5D scores) over time, comparing decompression and fusion within the VP and non-VP groups. There were no major complications observed in either group. In the endoscopic decompression group, no revision surgeries were required. One case of revision due to hematoma occurred in the non-VP group. Additionally, incidental durotomies were noted in one patient from the non-VP group and two patients from the VP group. During OLIF surgery in the VP group, an iliac vein branch injury occurred, but it was successfully repaired intraoperatively by a vascular surgeon without further complications. No revision surgeries were required in the OLIF group.

## 4. Representative Cases

**Figure 4:**
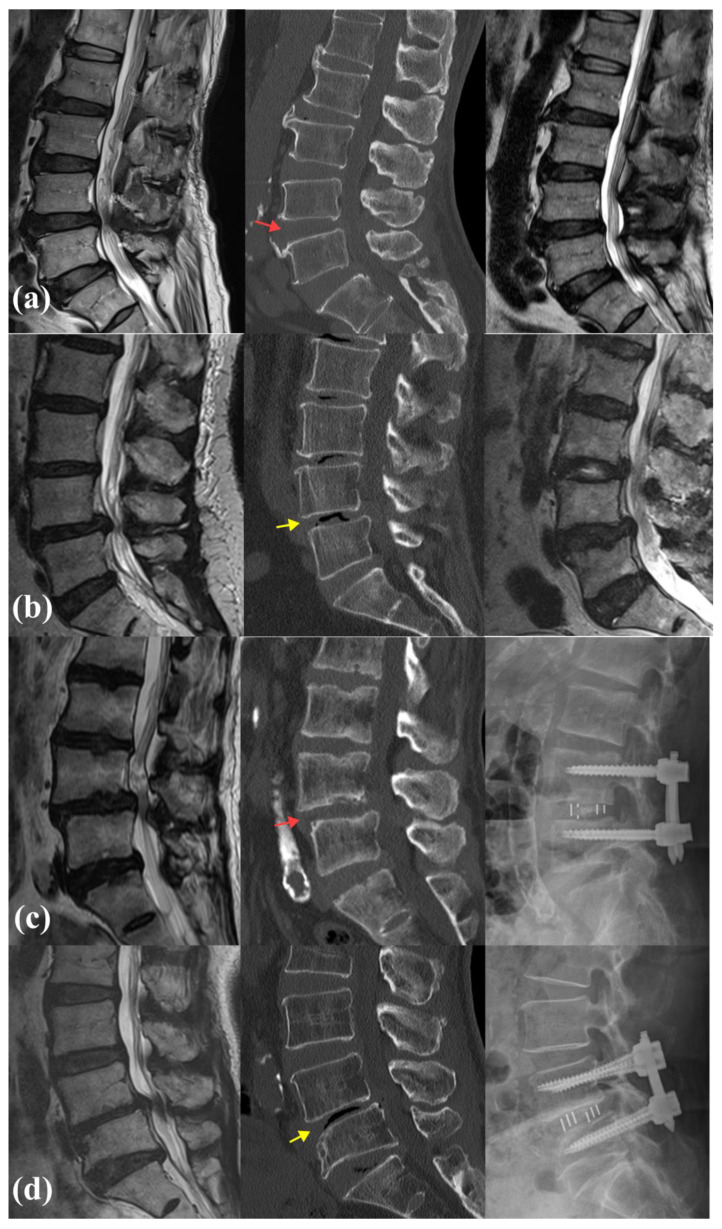
Comparative cases of patients with and without the VP undergoing endoscopic decompression and OLIF. (**a**) Preoperative MRI shows severe L4-5 stenosis with no VP observed on CT (non-VP, indicated by red arrow). Postoperative MRI confirms adequate decompression following endoscopic decompression. (**b**) Preoperative MRI shows severe L4-5 stenosis, with Grade 2 VP detected on CT (VP sign indicated by yellow arrow). Postoperative MRI confirms adequate decompression following endoscopic decompression. (**c**) Preoperative MRI reveals severe stenosis at L4-5 due to degenerative spondylolisthesis (DSL), but no significant VP sign is observed on CT (non-VP, indicated by red arrow). MIS OLIF was performed at L4-5. (**d**) Preoperative MRI shows severe stenosis at L4-5, with Grade 2 VP confirmed on CT (VP sign indicated by yellow arrow). MIS OLIF was performed at L4-5.5.

Figure 4 illustrates four representative cases comparing VP and non-VP patients treated with either endoscopic decompression or MIS OLIF. In Figure 4a, a patient with severe L4-5 stenosis and no evidence of the VP on CT underwent endoscopic decompression. In Figure 4b, a patient with Grade 2 VP at L4-5 also underwent endoscopic decompression. Figure 4c shows a case of degenerative spondylolisthesis at L4-5 without the VP, where the patient was treated with MIS OLIF. Lastly, in Figure 4d, a patient with Grade 2 VP at L4-5 underwent MIS OLIF for fusion. The red arrow indicates non-VP, while the yellow arrow marks the presence of the VP on the CT scans.

## 5. Discussion

This study highlights the significant role of the disc VP in influencing the surgical outcomes of patients with lumbar spinal stenosis. Our study demonstrated that the patients in the VP group experienced more severe preoperative leg pain than those in the non-VP group. This aligns with the existing literature that associates the VP with advanced degenerative changes and segmental instability, typically resulting in increased back pain and disability [6,9]. However, our study further revealed that the VP not only correlates with these symptoms but also has a profound impact on radiculopathy. Patients with the VP who underwent fusion had better long-term outcomes in terms of leg pain than those who underwent decompression alone, suggesting that the instability caused by the VP extends beyond the disc and affects the nerve roots, thereby exacerbating radiculopathy. This supports the notion that the VP is a critical factor in determining the appropriate surgical approach, particularly when considering the potential benefits of fusion for stabilizing the spine and alleviating nerve irritation [11].

The underlying mechanisms by which the VP contributes to radiculopathy and leg pain likely involve several factors. Instability, driven by the presence of the VP, is a key contributor. Gas accumulation within the disc space, a characteristic of the VP, indicates significant disc degeneration, which in turn leads to micromovement in the affected spinal segment [8,30,31]. These micromovements can result in abnormal mechanical loading and increased stress on adjacent neural structures, particularly the nerve roots [11]. Mechanical irritation of the nerve roots may directly cause radicular symptoms that manifest as leg pain. Additionally, the instability and micromovements associated with the VP can induce a pro-inflammatory state within the disc and surrounding tissues [32]. This inflammatory response is mediated by the release of cytokines and other inflammatory mediators that can further irritate the nerve roots and contribute to the chronicity and severity of radiculopathy [32,33]. These combined mechanical and biochemical factors underscore the significance of the VP in the pathophysiology of radiculopathy in lumbar spinal stenosis.

Despite the known associations between the VP and increased back pain or higher ODI scores reported in previous studies [6,12,19,34], our study did not find a significant relationship between the VP and these outcomes. One possible explanation is that chronic back pain in these patients may have been present for an extended period, leading to a level of pain and disability that was less responsive to surgical interventions such as fusion. While previous studies often highlight a strong correlation between the VP and worsened back pain or ODI scores [6,9,10,11,12,17,19,34], our findings suggest that the severity of the VP does not necessarily equate to more severe back pain preoperatively. This could be because although the VP may suggest instability, decompression alone may still be a feasible option in cases where the disc height has significantly decreased [4,6,8]. Thus, it is essential to consider an individual patient’s pain profile and the extent of disc degeneration when deciding on a suitable surgical approach. In addition to the VP, which indicates a level of instability that may benefit from fusion, our study found a higher incidence of endplate sclerosis in the VP group. This sclerosis likely contributes to a more favourable environment for fusion, particularly with techniques like OLIF [11,17,20,22]. Endplate sclerosis provides a solid bony interface, essential for safely inserting an OLIF cage and restoring disc height [20,21,22]. This suggests that the higher incidence of endplate sclerosis in the VP group could influence the decision to perform fusion rather than decompression.

However, our study challenges the notion that a higher VP grade is associated with increased back pain, as suggested by Ohyama et al. [34], who reported a direct correlation between the VP grade and back pain severity. Our findings indicate that the presence of the VP does not always translate into severe back pain, nor does it guarantee a poor surgical outcome. This highlights the need for a better understanding of the VP and its impact on clinical symptoms, suggesting that the VP may not be a poor indicator of surgical outcomes, as previously reported [10,11,19,34].

In contrast, in the absence of the VP, the differences in outcomes between decompression and fusion were minimal. This finding is consistent with previous studies suggesting that fusion may not be necessary in patients without the VP, where decompression alone might suffice [4,7,21,35]. Moreover, our results showed that at the 1-month follow-up, patients in the non-VP group who underwent decompression had significantly better ODI scores than those in the VP group who underwent fusion. This suggests that in cases without the VP, the less invasive nature of decompression combined with its ability to relieve neural compression directly can lead to better immediate outcomes and faster recovery [7,8,35]. These observations underscore the potential of the VP as a guideline for surgical decision-making, although it is not an absolute indication for fusion. This suggests that in the absence of the VP, decompression may be preferable, sparing the additional risks and recovery times associated with fusion [4,7,36,37].

The ongoing debate between fusion and decompression in the management of lumbar spinal stenosis is well documented. Traditionally, factors such as patient age, instability, and the degree of degenerative change have guided this decision [4,7,8,22,30,35,36]. Fusion is often favoured in cases of severe arthritic changes, instability, or listhesis, aiming to achieve solid arthrodesis and prevent further degeneration [4,36,37]. However, decompression alone is effective in patients without significant instability or those at a higher risk for complications associated with fusion [4,7,8]. Our study adds to this body of evidence by demonstrating that the VP, indicative of disc degeneration and instability, could be a valuable factor in guiding this choice. Specifically, our findings suggest that fusion may be more beneficial in patients with the VP, whereas those without the VP could potentially achieve outcomes similar to those opting for decompression alone.

Moreover, the VP and disc height relationship should not be overlooked, particularly in the context of minimally invasive procedures such as OLIF. OLIF is known for its ability to restore disc height and achieve indirect decompression, making it a suitable option for patients with the VP [20,21,22] in whom maintaining or restoring disc height is crucial. Conversely, in cases where the disc height is already significantly reduced, other factors such as ligamentum flavum hypertrophy and central canal stenosis become more critical, potentially influencing the decision to perform direct decompression [4]. These considerations highlight the need for a nuanced approach when selecting the appropriate surgical intervention, considering the presence of the VP and the overall spinal environment and pathologies.

Our study had some limitations. First, the relatively small sample size, particularly when subgrouping patients into the VP and non-VP groups, may limit the generalizability of our findings. This sample size constraint could influence the robustness of our results and therefore, further studies with larger cohorts are needed to validate these findings and strengthen their applicability in broader clinical practise. Secondly, although we used MIS OLIF and endoscopic decompression as representative surgical techniques, these methods may not fully capture the outcomes of traditional open fusion or decompression surgeries, respectively. Although MIS OLIF and endoscopic decompression are currently increasingly favoured owing to their minimally invasive nature [2,5,23], they may not be directly comparable to other more conventional procedures. This limits the applicability of the results to all fusion and decompression surgeries. However, the growing adoption of these minimally invasive approaches in clinical practise underscores the relevance of our study in addressing current surgical trends [23]. Finally, while our study identifies the VP as a potential factor in deciding between decompression and fusion, it does not provide a definitive algorithm for surgical decision-making. To develop such a strategy, it would be necessary to integrate and validate additional measures of instability, such as the degree of listhesis, facet joint cystic changes, and dynamic radiographic findings. Further research could use our findings to inform future guidelines, with the VP being one of the important factors in the overall assessment of instability.

## 6. Conclusions

Although the presence of the VP should not be viewed as an absolute indication for fusion, it may play a considerable role in determining surgical outcomes, particularly leg pain and radiculopathy. Our findings suggest that the presence of the VP may be related to leg pain recurrence when fusion is not performed. Further large cohort studies or randomized controlled trials are required to confirm our findings and thoroughly explore the implications of the VP in spinal surgery.

## Figures and Tables

**Table 1:** Comparison of demographic characteristics between VP and non-VP groups.

	Non-VP	VP	*p* Value
	(n = 42)	(n = 68)	
Age, years	67.5 ± 11.2	70.4 ± 9.0	0.203
Sex, M:F	19:23	40:28	0.165
ASA classification			0.569
2	21 (60.0%)	24 (52.2%)	
3	14 (40.0%)	21 (45.7%)	
4	0 (0.0%)	1 (2.2%)	
Height, cm	159.8 ± 5.7	157.2 ± 7.3	0.137
Weight, kg	62.5 ± 11.3	60.7 ± 8.5	0.208
BMI (kg/m^2^)	24.5 ± 2.4	24.6 ± 3.3	0.975
BMD, T-score	−1.2 ± 1.8	−1.5 ± 1.4	0.702
HTN, n	15 (35.7%)	21 (30.9%)	0.599
DM, n	7 (16.7%)	17 (25.0%)	0.304
Smoking, n	5 (11.9%)	11 (16.2%)	0.537
Operation type			0.556
Decompression	23 (54.8%)	32 (47.1%)	
Fusion	19 (45.2%)	36 (52.9%)	
Location			0.616
L1–2	0 (0.0%)	1 (1.5%)	
L2–3	1 (2.4%)	6 (8.8%)	
L3–4	5 (11.9%)	9 (13.2%)	
L4–5	31 (73.8%)	44 (64.7%)	
L5–S1	5 (11.9%)	8 (11.8%)	

VP, vacuum phenomenon; ASA, American Society of Anesthesiologists physical status classification; BMD, bone mineral density; HTN, hypertension; DM, diabetes mellitus.

**Table 2:** Comparison of imaging characteristics between VP and non-VP groups.

	Non-VP	VP	*p* Value
	(n = 42)	(n = 68)	
Schizas grade, n			0.773
B	2	3	
C	23	41	
D	18	24	
VP grade, n			<0.001 *
0	42 (100.0%)		
1		27 (39.7%)	
2		29 (42.6%)	
3		12 (17.6%)	
Endplate sclerosis, n	6 (14.3%)	33 (48.5%)	0.001 *
ISL, n	2 (4.8%)	6 (8.8%)	0.425
CT measurements			
ADH, mm	9.7 ± 2.9	7.6 ± 2.8	<0.001 *
PDH, mm	5.5 ± 1.9	3.8 ± 1.4	<0.001 *
RFH, mm	11.6 ± 2.9	10.9 ± 2.5	0.262
RFA, mm^2^	67.0 ± 22.5	61.4 ± 21.1	0.222
LFH, mm	11.8 ± 2.8	12.2 ± 9.4	0.762
LFA, mm^2^	66.2 ± 21.0	64.5 ± 18.4	0.673

VP, vacuum phenomenon; ISL, isthmic spondylolisthesis; ADH, anterior disc height; PDH, posterior disc height; RFH, right foraminal height; RFA, right foraminal area; LFH, left foraminal height; LFA, left foraminal area. * *p* value < 0.05.

**Table 3:** Clinical measures preoperatively and at the final follow-up for VP and non-VP groups.

	Total	Non-VP	VP	*p* Value
	(n = 110)	(n = 42)	(n = 68)	
Preoperative				
Back pain	6.5 ± 2.3	6.3 ± 2.5	6.7 ± 2.0	0.55
Leg pain	4.8 ± 2.0	4.4 ± 1.8	5.3 ± 2.1	0.010 *
ODI score	61.1 ±15.8	59.7 ± 15.9	62.5 ± 15.9	0.335
Eq5D score	15.4 ± 3.1	15.4 ± 3.3	15.4 ± 2.8	0.856
2 years				
Back pain	2.3 ± 1.9	2.3 ± 2.0	2.3 ± 1.9	0.948
Leg pain	1.4 ± 1.5	1.3 ± 1.4	1.6 ± 1.6	0.422
ODI score	29.8 ± 17.2	25.8 ± 17.5	32.6 ± 16.6	0.085
Eq5D score	9.1 ± 3.3	8.8 ± 3.5	9.4 ± 3.2	0.449

VP, vacuum phenomenon; ODI, Oswestry Disability Index; Eq5D, EuroQol-5 Dimension. * *p* value < 0.05.

**Table 4:** Comparison of clinical outcomes between decompression and fusion in non-VP patients.

	Decompression (n = 23)	Fusion (n = 19)	*p* Value
Back Pain VAS			
Preoperative	6.6 ± 2.0	6.4 ± 2.2	0.738
1 Month	2.7 ± 1.5	2.7 ± 2.0	0.9
6 Months	2.0 ± 1.6	2.2 ± 2.0	0.751
1 Year	2.3 ± 1.8	2.2 ± 1.7	0.832
2 Years	2.1 ± 1.8	2.6 ± 2.3	0.499
Leg Pain VAS			
Preoperative	4.0 ± 2.1	4.7 ± 1.9	0.293
1 Month	1.9 ± 1.4	1.9 ± 2.2	0.974
6 Months	1.5 ± 1.7	1.0 ± 1.4	0.29
1 Year	1.1 ± 1.0	1.0 ± 1.2	0.684
2 Years	1.3 ± 1.1	1.3 ± 1.7	0.964
Oswestry Disability Index			
Preoperative	61.6 ± 17.5	64.6 ± 12.5	0.55
1 Month	33.0 ± 13.9	44.3 ± 15.6	0.018 *
6 Months	24.4 ± 10.3	31.9 ± 17.6	0.135
1 Year	23.0 ± 12.0	29.8 ± 17.1	0.22
2 Years	22.5 ± 14.4	29.8 ± 20.4	0.242
EuroQol-5 Dimension			
Preoperative	14.8 ± 3.4	15.2 ± 2.9	0.695
1 Month	9.7 ± 2.6	10.8 ± 2.9	0.24
3 Months	8.5 ± 2.5	9.0 ± 3.0	0.595
6 Months	8.0 ± 1.8	9.2 ± 3.3	0.235
1 Year	8.2 ± 2.4	9.5 ± 4.5	0.35

VAS, visual analogue scale; VP, vacuum phenomenon. * *p* value < 0.05.

**Table 5:** Comparison of clinical outcomes between decompression and fusion in the VP group.

	Decompression (n = 32)	Fusion (n = 36)	*p* Value
Back Pain VAS			
Preoperative	6.7 ± 2.1	7.0 ± 1.6	0.499
1 Month	2.5 ± 1.5	3.5 ± 1.9	0.02
6 Months	2.5 ± 2.2	2.5 ± 1.3	0.973
1 Year	2.3 ± 1.6	2.8 ± 1.3	0.24
2 Years	2.7 ± 2.5	2.0 ± 1.2	0.27
Leg Pain VAS			
Preoperative	5.2 ± 2.2	5.2 ± 2.1	1
1 Month	2.2 ± 1.8	2.7 ± 1.3	0.355
6 Months	1.7 ± 1.6	1.8 ± 1.7	0.664
1 Year	1.9 ± 1.5	1.3 ± 1.5	0.042 *
2 Years	2.1 ± 1.8	1.2 ± 1.4	0.017 *
Oswestry Disability Index			
Preoperative	59.7 ± 13.8	60.0 ± 12.7	0.939
1 Month	38.5 ± 17.2	46.4 ± 10.2	0.028
6 Months	32.3 ± 20.7	36.2 ± 12.6	0.382
1 Year	28.7 ± 17.4	34.4 ± 13.1	0.196
2 Years	34.4 ± 21.7	31.0 ± 11.0	0.52
EuroQol-5 Dimension			
Preoperative	15.1 ± 2.3	15.4 ± 2.7	0.726
1 Month	10.0 ± 2.7	11.3 ± 2.3	0.079
6 Months	9.9 ± 3.8	9.9 ± 1.9	0.967
1 Year	9.0 ± 3.3	9.7 ± 2.0	0.423
2 Years	9.6 ± 4.3	9.2 ± 2.2	0.723

VAS, visual analogue scale; VP, vacuum phenomenon. * *p* value < 0.05.

## Data Availability

The datasets used and/or analyzed in the current study are available from the corresponding author upon reasonable request.
